# Lung Ultrasound Reproducibly Outperforms Computed Tomography in the Detection of Extravascular Lung Water in Patients Undergoing Haemodialysis

**DOI:** 10.3390/diagnostics14060589

**Published:** 2024-03-11

**Authors:** John P. Corcoran, Mark Hew, Ben Attwood, Murali Shyamsundar, Sheera Sutherland, Kristine Ventura, Rachel Benamore, Victoria St. Noble, Hania E. Piotrowska, Christopher W. Pugh, Christian B. Laursen, Fergus V. Gleeson, Najib M. Rahman

**Affiliations:** 1 Oxford Centre for Respiratory Medicine, Oxford University Hospitals NHS Foundation Trust, Oxford OX3 9DU, UK; najib.rahman@ndm.ox.ac.uk; 2 Oxford Respiratory Trials Unit, University of Oxford, Oxford OX3 7LE, UK; 3 Allergy, Asthma & Clinical Immunology, The Alfred Hospital, Melbourne, VIC 3004, Australia; 4 School of Public Health & Preventive Medicine, Monash University, Melbourne, VIC 3004, Australia; 5 Department of Anaesthesia and Intensive Care, South Warwickshire NHS Foundation Trust, Warwick CV34 5BW, UK; 6 Wellcome Wolfson Institute for Experimental Medicine, Queen’s University Belfast, Belfast BT7 1NN, UK; 7 Oxford Kidney Unit, Oxford University Hospitals NHS Foundation Trust, Oxford OX3 9DU, UK; 8 Department of Radiology, Oxford University Hospitals NHS Foundation Trust, Oxford OX3 9DU, UK; 9 Nuffield Department of Medicine, University of Oxford, Oxford OX3 7LE, UK; 10 Department of Respiratory Medicine, Odense University Hospital, 5000 Odense, Denmark; 11 Department of Clinical Research, University of Southern Denmark, 5000 Odense, Denmark; 12 NIHR Oxford Biomedical Research Centre, University of Oxford, Oxford OX3 7LE, UK

**Keywords:** lung ultrasound, extravascular lung water, interstitial syndrome

## Abstract

Background: Lung ultrasound (LUS) is increasingly used as an extension of physical examination, informing clinical diagnosis, and decision making. There is particular interest in the assessment of patients with pulmonary congestion and extravascular lung water, although gaps remain in the evidence base underpinning this practice as a result of the limited evaluation of its inter-rater reliability and comparison with more established radiologic tests. Methods: 30 patients undergoing haemodialysis were prospectively recruited to an observational cohort study (NCT01949402). Patients underwent standardised LUS assessment before, during and after haemodialysis; their total LUS B-line score was generated, alongside a binary label of whether appearances were consistent with an interstitial syndrome. LUS video clips were recorded and independently scored by two blinded expert clinician sonographers. Low-dose non-contrast thoracic CT, pre- and post dialysis, was used as a “gold standard” radiologic comparison. Results: LUS detected a progressive reduction in B-line scores in almost all patients undergoing haemodialysis, correlating with the volume of fluid removed once individuals with no or minimal B-lines upon pre-dialysis examination were discounted. When comparing CT scans pre- and post dialysis, radiologic evidence of the change in fluid status was only identified in a single patient. Conclusions: This is the first study to demonstrate that LUS detects changes in extravascular lung water caused by changing fluid status during haemodialysis using a blinded outcome assessment and that LUS appears to be more sensitive than CT for this purpose. Further research is needed to better understand the role of LUS in this and similar patient populations, with the aim of improving clinical care and outcomes.

## 1. Introduction

Ultrasound has been used as a diagnostic tool in medicine for over sixty years, but its decentralisation from radiology departments and into the hands of clinicians at the patient’s bedside is a recent development, facilitated by changing technology as machines become more portable without compromising their image quality. Increased familiarity with and access to ultrasound, alongside a recognition of the limitations of conventional physical examination and chest X-ray, have encouraged clinicians to explore its use as a diagnostic tool [1], to the point that ultrasound is now as essential a tool as the stethoscope in managing patients with respiratory disease [2].

Lung ultrasound (LUS) has delivered a paradigm shift in the assessment of patients with pulmonary congestion and excess extravascular lung water (EVLW), using the presence, number, and pattern of B-lines on LUS as a surrogate marker for pulmonary oedema [3,4]. Despite published consensus guidelines on the use of point-of-care LUS [5], gaps remain in the evidence base and physiological data underpinning this clinical practice. Previous work [6,7] has used patients with end-stage renal failure, requiring haemodialysis, as a population with predictable fluid overload to explore the relationship between changing fluid status and the presence of EVLW, and how this can be seen on LUS. However, these studies have suffered from a lack of a blinded LUS assessment, an evaluation of inter-rater reliability, and/or a radiologic gold-standard reference test; issues often encountered when studying diagnostic LUS [8].

This pilot study evaluated the ability of LUS to diagnose changes in EVLW over time, utilizing a stable outpatient population under close physiological observation. This study built on the existing evidence base, firstly by facilitating a direct comparison with the existing gold-standard radiologic test for lung parenchymal assessment (i.e., computed tomography), and secondly by using multiple assessors blinded to study participants’ clinical status to evaluate the consistency of LUS interpretations between different operators.

## 2. Methods

### 2.1. Study Design and Participants

This was a prospective observational cohort study, recruiting patients from a single site over a 15-month period. Adult patients (≥18 years old) were screened from normal clinical care at the outpatient haemodialysis unit by the clinicians responsible for this service. Patients were excluded if they were <18 years old, unable to provide informed consent, or either pregnant or breastfeeding. Study participants provided informed written consent before recruitment and study-related procedures.

The study was sponsored by the University of Oxford, managed through the University of Oxford Respiratory Trials Unit, and registered with ClinicalTrials.gov (NCT01949402). The protocol and its subsequent amendments were approved by the UK National Research Ethics Service (13/SC/0319). This study was supported by the Teresa Rosenbaum Golden Charitable Trust (Rosetrees Trust), who provided a grant to cover administrative and consumable costs, and by Esaote UK, who provided ultrasound equipment. The funders were not involved in the study’s design or procedures, data analysis or interpretation, or manuscript production.

### 2.2. Study Procedures

All study procedures were carried out during a single hospital visit that was coordinated with one of the participant’s regular haemodialysis sessions to minimise any disruption to usual clinical care. Haemodialysis was performed using a B. Braun Dialog Plus machine (B. Braun Medical, Melsungen, Germany) with a dry weight target set in advance by the participant’s responsible renal physician. Baseline demographic data and physical observations (including the volume of fluid removed by the haemodialysis machine at that timepoint) before, during, and after haemodialysis were recorded; a full blood count (including haematocrit) was also performed pre- and post dialysis.

Study participants underwent standardised LUS examination with an Esaote MyLab25 Gold machine (Esaote UK, Cambridge, UK) and an abdominal curvilinear transducer (3–6 MHz) immediately before, midway through, and immediately after haemodialysis. A modified abdominal preset was used, with all smoothing and artefact minimisation algorithms switched off prior to image acquisition, with the probe placed along the intercostal space. LUS clips (6–10 s in duration, including at least two full respiratory cycles) were acquired, with the patient sitting up at between 45 and 90 degrees according to comfort, at 10 points over each hemithorax (Supplementary Materials Figure S1) and reported in real time by the primary (bedside) operator. The same LUS video clips were remotely scored at a later stage by two independent assessors blinded to the original scoring and the participants’ clinical status. All LUS scorers were expert practitioners who use LUS regularly as part of their usual clinical practice and have previously published in this area of research. Video clips from different patients and different timepoints were presented to the LUS scorers in a random order, and they were asked to document the maximum number of B-lines seen at each of the 10 points over each hemithorax at any given time during the video clip; these were then added together in order to generate a total LUS B-line score for the patient at that timepoint. B-lines were pre-defined as echogenic, dynamic, wedge-shaped artefacts with a narrow base originating at the pleural line and extending to the distal edge of the ultrasound image, consistent with the previous literature [3,5]. All LUS scorers were asked to decide whether the overall examination findings were consistent with an interstitial syndrome as defined in the consensus guidelines [5].

Study participants underwent non-contrast CT of their chest before and after haemodialysis, performed with the participant lying supine, using a 16-slice scanner (GE Lightspeed; GE Healthcare, Buckinghamshire, UK) with a slice thickness of 0.625 mm. Images were acquired during inspiratory breath hold to minimize motion artefacts while using a low-dose protocol (maximum 1.7 mSv). CT scans were reported by a consultant radiologist with a sub-specialty interest in thoracic imaging. The reporting radiologist was not blinded to the participant’s clinical condition or timing of the scan and was specifically instructed to look for changes that would be suggestive of a change in fluid status.

Study participants completed a questionnaire (Supplementary Materials File S1) relating to their symptoms pre- and post dialysis, and their experience of and satisfaction with the LUS assessment. This included a visual analogue scale (VAS) score of the pain caused by LUS examination (0–100 mm: no pain at 0 mm and worst possible pain at 100 mm); VAS (0–100 mm: no breathlessness at 0 mm and worst possible breathlessness at 100 mm) and Likert-type scores of breathlessness pre- and post dialysis; and an assessment of their willingness to undergo LUS examination again in the future.

### 2.3. Outcomes and Analyses

The primary outcome measure was the correlation between the change in the total LUS B-line score, taken to represent the presence and extent of EVLW, and the contemporaneous associated change in fluid status during haemodialysis. Fluid status was assessed using physiological markers, including body weight and haematocrit pre- and post dialysis; the volume of fluid removed during dialysis was measured by the haemodialysis machine.

The secondary outcome measures were (a) change in the total LUS B-line score and change in patient-reported breathlessness, measured using visual analogue and Likert-type scales; (b) measurement of patient comfort and satisfaction with the LUS examination using visual analogue and Likert-type scales; and (c) comparison of the total LUS B-line score and the categorical diagnosis of an interstitial syndrome, made using pre-defined criteria [5], ascribed to individual ultrasound scans by different operators.

SPSS version 24 was used for statistical analyses; a *p*-value < 0.05 was considered significant. Descriptive statistics were used to summarize patient characteristics. *T*-tests were used to analyse parametric data. The Pearson correlation coefficient was used to assess the linear correlation between different variables. Interrater reliability was calculated using kappa statistics for categorical data; for continuous variables, the intraclass correlation coefficient was calculated to estimate the degree of their association, alongside Bland–Altman analysis [9] to demonstrate individual differences between measures. 

## 3. Results

### 3.1. Baseline Data

Thirty participants were recruited; their baseline characteristics are in Table 1. All participants completed haemodialysis sessions (between 3 and 4 h in duration, depending on clinical need) and all study-related procedures without complications.

**Figure 1:**
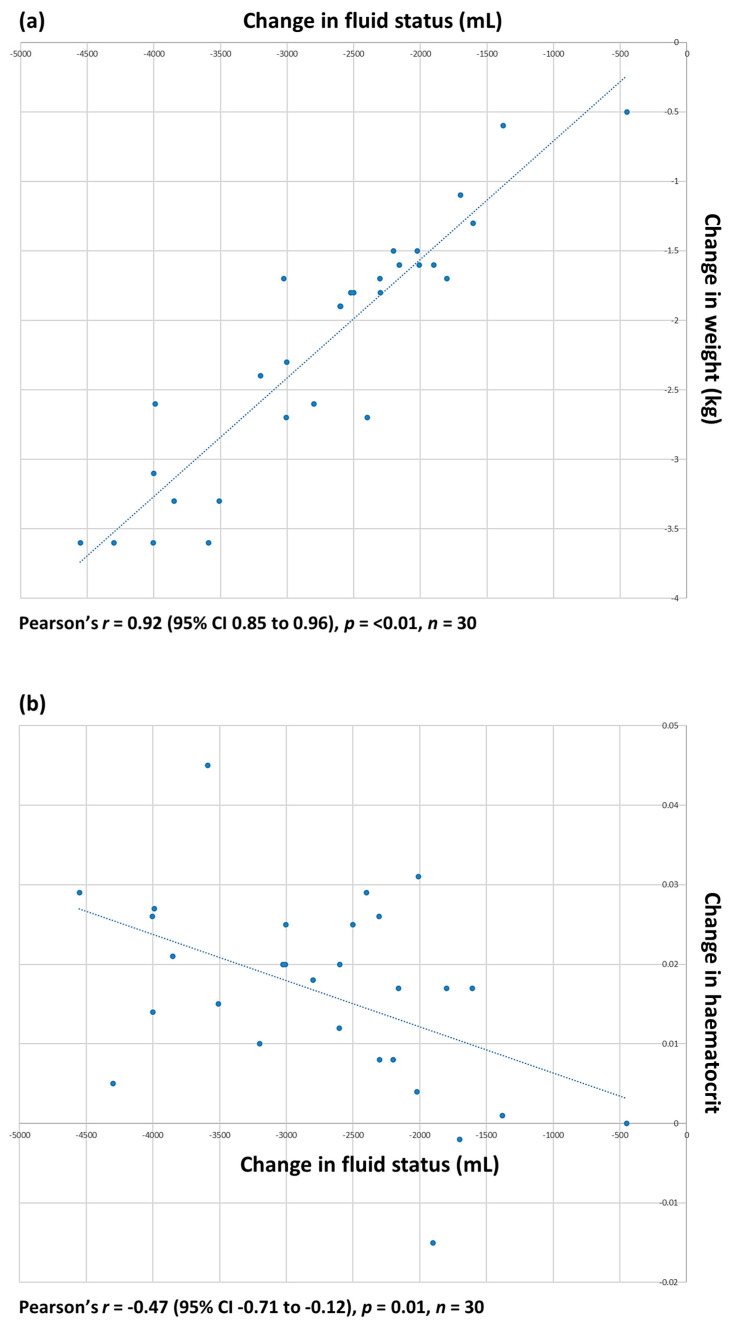
Relationship between (**a**) change in weight, kg, pre- and post dialysis and volume of fluid, mL, removed during haemodialysis; and (**b**) change in haematocrit pre- and post dialysis and volume of fluid removed.

Data assessing the relationship between the measurements used in normal clinical practice to monitor the changes in fluid status during haemodialysis showed an extremely strong positive correlation between the change in patients’ weight pre- and post haemodialysis and the volume of fluid removed during haemodialysis, as measured by the machine. There was a weaker negative correlation between the change in haematocrit pre- and post haemodialysis and the volume of fluid removed during haemodialysis (Figure 1).

### 3.2. Primary Endpoint

**Figure 2:**
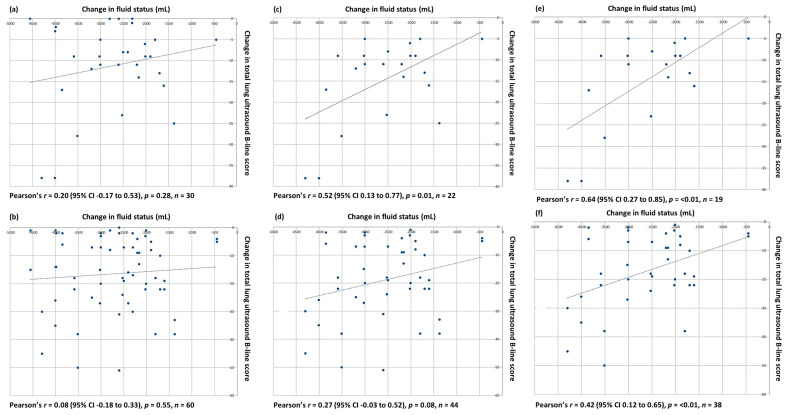
Relationship between volume of fluid, mL, removed during haemodialysis and change in total LUS B-line score for all study participants: (**a**) primary scorer, (**b**) blinded scorers; excluding participants with minimal B-lines on their baseline ultrasound: (**c**) primary scorer, (**d**) blinded scorers; and excluding participants with minimal B-lines and/or ILD: (**e**) primary scorer, (**f**) blinded scorers.

An initial analysis using two timepoints (before and after haemodialysis) did not show any correlation between the reduction in the total LUS B-line score recorded by the primary LUS operator and the volume of fluid removed during haemodialysis (Figure 2a,b). This analysis included several study participants who had a minimal number of B-lines prior to haemodialysis and thus no sonographic evidence of excess EVLW that could be measured. The data were therefore re-analysed, this time excluding study participants who had minimal B-lines on their baseline (pre-dialysis) LUS scan. The exclusion criteria were based on a version of the sonographic categorisation of EVLW accumulation used in prior work [7,10], modified to reflect this study’s use of a 20-point (vs. 28-point) LUS protocol. On this basis, 8/30 (26.7%) study participants with a total LUS B-line score of 10 or below at baseline, as determined by at least two of the three LUS scorers, were considered to have no or minimal EVLW and were therefore excluded. Analysis of the LUS data from the remaining 22 (73.3%) participants demonstrated at least a moderate positive correlation between the reduction in their total LUS B-line score and the volume of fluid removed during haemodialysis (Figure 2c,d). 

In total, 3/30 (10%) participants had concomitant interstitial lung disease (ILD), recognised as a potential confounder in the assessment of B-lines on an LUS. None of the patients were known to have ILD prior to recruitment, with the diagnosis only made during this study’s CT. A reduction in total LUS B-line scores was still seen in these three participants by all LUS scorers; however, when they were excluded from the data analysis the correlation between the reduction in the total LUS B-line score and volume of fluid removed during haemodialysis improved for the remaining study participants (Figure 2e,f).

**Figure 3:**
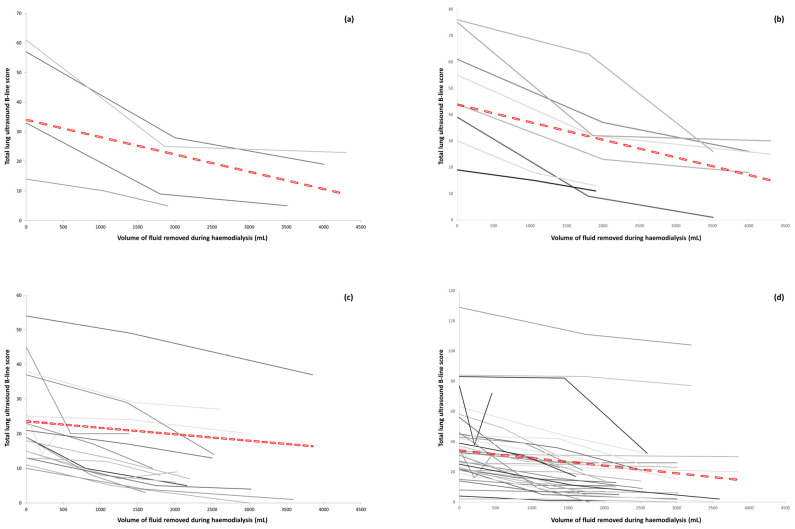
Change in total LUS B-line score vs. volume of fluid, mL, removed during haemodialysis for study participants with CT evidence of fluid overload: (**a**) primary scorer, (**b**) blinded scorers; and no CT evidence of fluid overload: (**c**) primary scorer, (**d**) blinded scorers. Dashed lines represent the best fit for each group.

When analysing data from three timepoints (before, during, and after haemodialysis), there was a correlation between the changes in the total LUS B-line score and the volume of fluid removed, but this relationship was not linear (Figure 3). No study participants had an increase in their total LUS B-line score from pre- to post dialysis.

### 3.3. Comparison of LUS and CT Findings

In total, 4/30 (13.3%) participants were reported as having evidence of fluid overload on the pre-dialysis CT scan of their chest. In two cases this was reported as “clear fluid overload”, with features including cardiomegaly, small bilateral pleural effusions, and widespread interlobular septal thickening throughout the lung parenchyma. In two cases, these changes were reported as “subtle fluid overload”, with interlobular septal thickening favouring dependent areas of the lung parenchyma. The remaining 26/30 (86.7%) participants were reported as having no evidence of fluid overload on their pre-dialysis CT scan; this was unchanged on their post-dialysis CT scans.

Of the four participants with fluid overload on their pre-dialysis CT scans, only one (initially described as “clear fluid overload”) was reported as showing any evidence of improvement on their post-dialysis CT scan, with the resolution of one pleural effusion and the near-total clearance of their prior interlobular septal thickening. The other three participants with evidence of fluid overload on their pre-dialysis CT scans were reported as having no change in appearance in their post-dialysis CT scans. 

The four study participants with evidence of fluid overload on their pre-dialysis CT scans all had a reduction in their total LUS B-line score during haemodialysis (Figure 3a,b) that was observed by both primary and blinded LUS scorers. The mean absolute reduction in B-line scores was 28.3 (SD 13.7) for the primary scorer, and 31.1 (SD 14.0) for the blinded scorers. The mean percentage reduction from pre- to post dialysis was 69.5% (SD 10.4) for the primary scorer and 61.6% (SD 15.9) for the blinded scorers. 

For those study participants who had no evidence of fluid overload on their pre-dialysis CT, but who did have evidence of excess B-lines on their baseline LUS, as defined above (see Section 3.2), there was a reduction in their total LUS B-line scores during haemodialysis (Figure 3c,d), observed by both primary and blinded LUS scorers. The mean absolute reductions in B-line score were 11.6 (SD 5.8, primary scorer) and 16.5 (SD 12.1, blinded scorers), and the mean percentage reductions from pre- to post dialysis were 52.3% (SD 25.4, primary scorer) and 50.6% (SD 28.5, blinded scorers).

When directly comparing CT and LUS for both the detection of and the identification of post-dialysis changes in EVLW, CT was able to detect the initial presence of EVLW in 4/30 (13.3%) and the post-dialysis change in 1/30 (3.3%) participants. LUS was able to detect the initial presence of and post-dialysis changes in EVLW in 22/30 (73.3%) participants, whether assessed by the primary or blinded scorers.

### 3.4. Secondary Endpoints

#### 3.4.1. Correlation of Changes in Total LUS B-Line Scores with Patient-Reported Breathlessness

The patient-reported change in breathlessness following haemodialysis was measured using Likert-type and VAS scores, with a reduction in breathlessness of ≥10 mm on the VAS considered significant [11] in the absence of population-specific reference data. The mean change in the VAS breathlessness score following haemodialysis was −4.8 mm (SD 10.1; 95% CI −8.6 to −1.0), with 5/30 (16.7%) participants reporting a significant reduction in breathlessness according to the pre-specified threshold. A total of 11/30 (36.7%) participants reported an improvement in their breathing (4 slightly, 3 moderately, and 4 significantly) using the Likert-type scale.

The mean reduction in the total LUS B-line score for any study participant reporting an improvement in breathlessness following haemodialysis was 15.3 (SD 11.3; 95% CI 8.1 to 22.5), compared with 8.5 (SD 8.8; 95% CI 4.1 to 12.9) in those without an improvement in breathlessness, but this did not reach clinical significance (mean difference −6.8; 95% CI −14.3 to 0.8; *p* = 0.07, unpaired *t*-test).

#### 3.4.2. Patient Satisfaction with the LUS Examination

All participants reported their willingness to have the same LUS scans performed again in future if clinically appropriate. A total of 15/30 (50%) participants did not find the LUS examination time-consuming at all; 14/30 (46.7%) reported it to be slightly time-consuming; whilst 1/30 (3.3%) found it very time-consuming. The mean VAS pain score reported in association with the LUS examination was 4.8 mm (SD 5.9; 95% CI 2.6 to 7.0).

#### 3.4.3. Consistency of LUS Scoring between Clinicians

**Figure 4:**
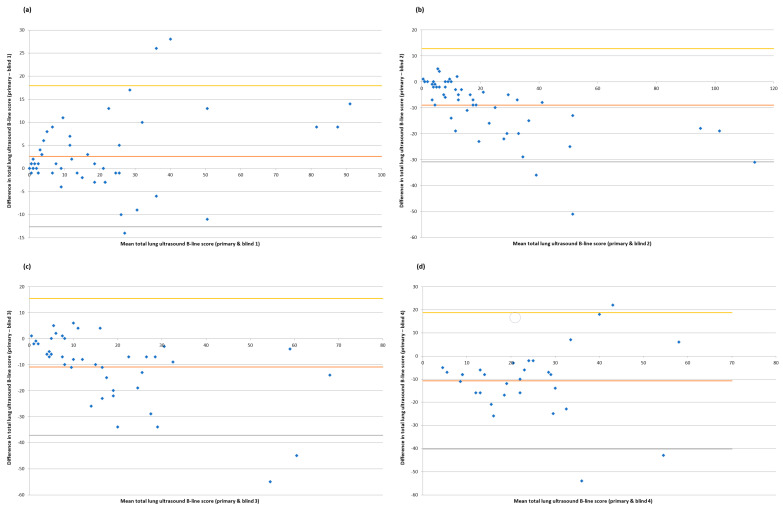
Bland–Altman plots assessing pairwise agreements for total lung ultrasound B-line scores (primary operator vs. blinded scorers 1–4). (**a**) Primary bedside operator vs. blind scorer 1; number of paired scans = 54; Pearson’s r = 0.94, *p* = <0.01; Bland-Altman analysis shows mean difference of 2.6 with 95% limits of agreement of −12.6 to 17.9. (**b**) Primary bedside operator vs. blind scorer 2; number of paired scans = 54; Pearson’s r = 0.94, *p* = <0.01; Bland-Altman analysis shows mean difference of −9.0 with 95% limits of agreement of −30.9 to 12.8. (**c**) Primary bedside operator vs. blind scorer 3; number of paired scans = 42; Pearson’s r = 0.78, *p* = <0.01; Bland-Altman analysis shows mean difference of −10.9 with 95% limits of agreement of −37.2 to 15.4. (**d**) Primary bedside operator vs. blind scorer 4; number of paired scans = 30; Pearson’s r = 0.53, *p* = <0.01; Bland-Altman analysis shows mean difference of −10.7 with 95% limits of agreement of −40.2 to 18.8.

Each LUS scan was scored by the primary operator and two independent clinicians blinded to the patient’s clinical condition and the time at which the LUS scan had been performed during haemodialysis. Inter-rater agreement for the diagnosis of an interstitial syndrome (a binary choice between present or absent) was moderate to good, with a free-marginal kappa of 0.61 (95% CI 0.47 to 0.73, *p* = <0.01) and 80% overall agreement between the three raters. Whilst scorers observed similar downward trends in the total LUS B-line scores of individual patients (Figure S2), with at least moderate inter-rater agreement as measured by a correlation coefficient, a Bland–Altman analysis demonstrated poor agreement between the precise total lung ultrasound B-line scores awarded by different raters to individual scans (Figure 4).

## 4. Discussion

This study demonstrates that LUSs can identify the presence of EVLW and monitor its resolution in real time, as evidenced by a reduction in the total LUS B-line scores observed during haemodialysis. We can be confident this is a genuine finding, since the change in fluid status incurred by haemodialysis was the only intervention that took place during the period of observation. This reduction in total B-line scores was observed by the primary LUS operator and also by independent blinded clinicians only given access to the scans, with no knowledge of either the patient’s physiological state or the timing of the imaging in relation to haemodialysis. The relationship between the change in the total B-line score and volume of fluid removed was not linear, suggesting that other factors have an influence on how the changes in intravascular volume status affect the extravascular fluid status of different individuals. The results are consistent with prior published data [6,7] and add to the evidence base by showing that LUS appears to outperform CT in both identifying lung changes that would be consistent with the presence of EVLW and showing the resolution of these same changes over time. 

Despite the LUS protocol requiring a 20-point examination, patients still found it acceptable. Minimal pain was reported with the LUS assessment, and all participants would have LUS scans in future if it were clinically necessary. An assessment of the change in patient-reported breathlessness following haemodialysis did not demonstrate an association between the reduction in total LUS B-line scores and improved symptoms; however, half the study population were not breathless before dialysis, with 15/30 (50%) participants reporting a pre-dialysis VAS breathlessness score of 10 mm or less. Despite this, there appeared to be a signal for the reduction in total B-line score to predict an improvement in breathing that merits evaluation in a larger study of more symptomatic patients. LUS changes may precede symptoms, and its use as a screening tool in this and other populations prone to the development of EVLW should be explored in future studies.

A major strength of this study was the use of blinded scorers, since LUS findings can be vulnerable to an individual operator’s ability to acquire and interpret images. Some tests of inter-rater reliability were reassuring, with 80% agreement of the diagnosis of interstitial syndromes and at least a moderate correlation between the primary operator and blinded scorers for total LUS B-line scores across different timepoints. However, an evaluation of the absolute agreement between raters on individual LUS scores using a Bland–Altman analysis demonstrated a lack of consistency. This is an important observation, since it suggests that, whilst the broader trends observed in LUSs during dialysis are common between different raters, the precise approach to scoring is unique to each individual clinician (Figure 4 and Figure S2), suggesting an element of internal calibration as to what is normal (or abnormal) in an ultrasound assessment. This means that if LUS is used to monitor the change in EVLW over time, the findings may only be valid if the same assessor carries out each examination. It is worth noting that other studies [7,12] have demonstrated better inter-rater reliability for LUS examinations than in our work. One explanation for this may be our use of three scorers for each examination as opposed to two [7,12], which inevitably increases the likelihood of variations in scoring being identified. Another reason may be that we chose to look not only at the extremes of LUS examination—that is, pre- and post dialysis in this scenario—but also the area in between, represented by the LUS examination mid-dialysis in this study. We do not, however, feel that this difference with other previous work makes our findings any less valid; rather, they illustrate the subjectivity and nuance involved in what is a human process. Standardizing how LUS is used in different clinical situations [13] may offer some benefits in this regard, whilst further studies on artificial intelligence and computer-aided scoring may also help resolve this issue of human subjectivity over time [14,15]. Until then, clinicians will need to remain aware of the limitations of LUS in this specific context.

This study’s finding that LUS outperforms CT in the evaluation of EVLW is a novel one that raises several questions. The physiological and anatomical changes that underpin the development of B-lines on LUS are incompletely understood, although it was a study comparing LUS with CT that first associated their formation with increased subpleural interlobular septal thickening, secondary to either pulmonary oedema or fibrotic lung disease [3]. It has been theorized that, as lung water increases, the difference in acoustic impedance between the aerated lobular parenchyma and fluid-filled interstitium creates a highly reflective interface, a reverberation of ultrasound waves, and B-lines on LUSs.

During this study, in the majority of patients who were observed to have an excess of B-lines on their LUSs that resolved during haemodialysis, no correlating abnormality (either initial, or evolving between pre- and post-dialysis imaging) could be identified on CT scans, despite these scans being reported by unblinded radiologists with sub-specialist expertise in thoracic imaging. This suggests a change in lung anatomy and physiology either beyond the level of the interlobular septa or the resolution of CT, but one which remains detectable on LUS. This finding will need to be replicated and evaluated in greater detail in future studies, but it appears to suggest that, in this clinical context at least, LUS may now be considered the radiologic gold standard. 

The findings of this and previous studies on LUS in this patient population have potential implications for clinical practice. Chronic volume expansion in patients with end-stage renal failure may develop insidiously and is associated with increased long-term morbidity and mortality [16,17]. LUS surveillance may allow for the identification of patients with features of pulmonary congestion before they develop symptoms or physiological decompensation, and thus allow for earlier therapeutic intervention. Similar LUS techniques could be applied in the management of other conditions where fluid overload and pulmonary congestion are common; for example, patients with congestive cardiac failure [18]. In all these scenarios, well-designed and robust clinical trials are needed to demonstrate that LUS can modify hard clinical outcomes of relevance to patients and clinicians before its wider uptake can be recommended [19]. 

There were limitations to the design and delivery of this study. The population was recruited from a single centre with experience in the performance of clinical studies in both renal and respiratory medicine, and several patients did not have any radiologic evidence of excess EVLW prior to commencing haemodialysis. This meant that the number of cases included in the final analysis was relatively small and may have impacted our findings. Whilst the patients acted as their own controls and the changes across LUS examinations could therefore be reliably attributed to changing fluid status alone, it is uncertain what impact other co-morbidities might have; for example, how does significant left and/or right ventricular dysfunction impact on B-line resolution during haemodialysis? These questions would need to be addressed as part of a larger multi-centre study, which might also allow for an assessment of whether a more limited LUS protocol provides sufficient information to inform clinical decision making. All the scans were acquired by a single experienced LUS operator; future work should build on the robust methodology of using multiple blinded LUS scorers by having different bedside operators examine patients to ensure the reproducibility of findings at the point of acquisition. 

In conclusion, these results demonstrate that LUS reliably identifies the presence of and changes in EVLW in patients with end-stage renal failure undergoing haemodialysis. The absolute scoring of B-lines on LUS scans appears to be dependent on the individual operator, whereas the assessment of change is consistent across different clinicians. LUS appears more sensitive than CT at detecting changes in EVLW and may be considered the radiologic gold standard. These observations have implications for clinical care, and further research is needed to better characterize the role that LUS might play in the management of patients on haemodialysis and patients with other conditions that result in pulmonary congestion, with the long-term aim of reducing morbidity and mortality in these different populations. 

## Figures and Tables

**Table 1:** Baseline characteristics of the study population (total *n* = 30 participants); data are presented as either *n* (%age) or median (IQR).

**Sex**	Male	22 (73.3%)
	Female	8 (26.7%)
**Age, years**		63.6 (52.8–76.4)
**Aetiology of ESRF**	Diabetes	9
	Hypertension	4
	Adult polycystic kidney disease	4
	IgA nephropathy	3
	Autoimmune vasculitis	2
	Postinfectious GN	2
	Obstructive uropathy	2
	Idiopathic/unknown	2
	Mesangial proliferative GN	1
	Nephrectomy (malignancy)	1
**Other medical history**	COPD	12 (40%)
	Interstitial lung disease	3 (10%)
	Cardiac failure	6 (20%)
**Fluid removed during dialysis, mL**		2562 (2019–3531)

Abbreviations: IQR = interquartile range; ESRF = end-stage renal failure; IgA = Immunoglobulin A; GN = glomerulonephritis; COPD = chronic obstructive pulmonary disease.

## Data Availability

The data presented in this study are available on request from the corresponding author. The data are not publicly available due to the size and nature of the files (CT and ultrasound images) analysed for this study.

## Supplementary Materials

The following supporting information can be downloaded at: https://www.mdpi.com/article/10.3390/diagnostics14060589/s1. Supplement File S1. Participant VAS LUS Questionnaire. Figure S1. LUS exam protocol. Figure S2. Individual patient data (n = 22; excludes patients with minimal B-lines on baseline (pre-dialysis) ultrasound) showing change in total B-line score (y-axis) vs. fluid removed during dialysis (x-axis) with similar trends but variation in absolute scores from different scorers. (* patients with evidence of fluid overload on pre-dialysis CT scan; # patients with interstitial lung disease).
