# Supplementary material for: Lung Ultrasound Reproducibly Outperforms Computed Tomography in the Detection of Extravascular Lung Water in Patients Undergoing Haemodialysis
Source: Diagnostics (Basel). 2024 Mar 11;14(6):589. doi: 10.3390/diagnostics14060589 (PMC10969609; doi:10.3390/diagnostics14060589)

Figure S2: Individual patient data (n = 22; excludes patients with minimal B-lines on baseline (pre-dialysis) ultrasound) showing change in total B-line score (y-axis) vs. fluid removed during dialysis (x-axis) with similar trends but variation in absolute scores from different scorers.
(* patients with evidence of fluid overload on pre-dialysis CT scan; # patients with interstitial lung disease)

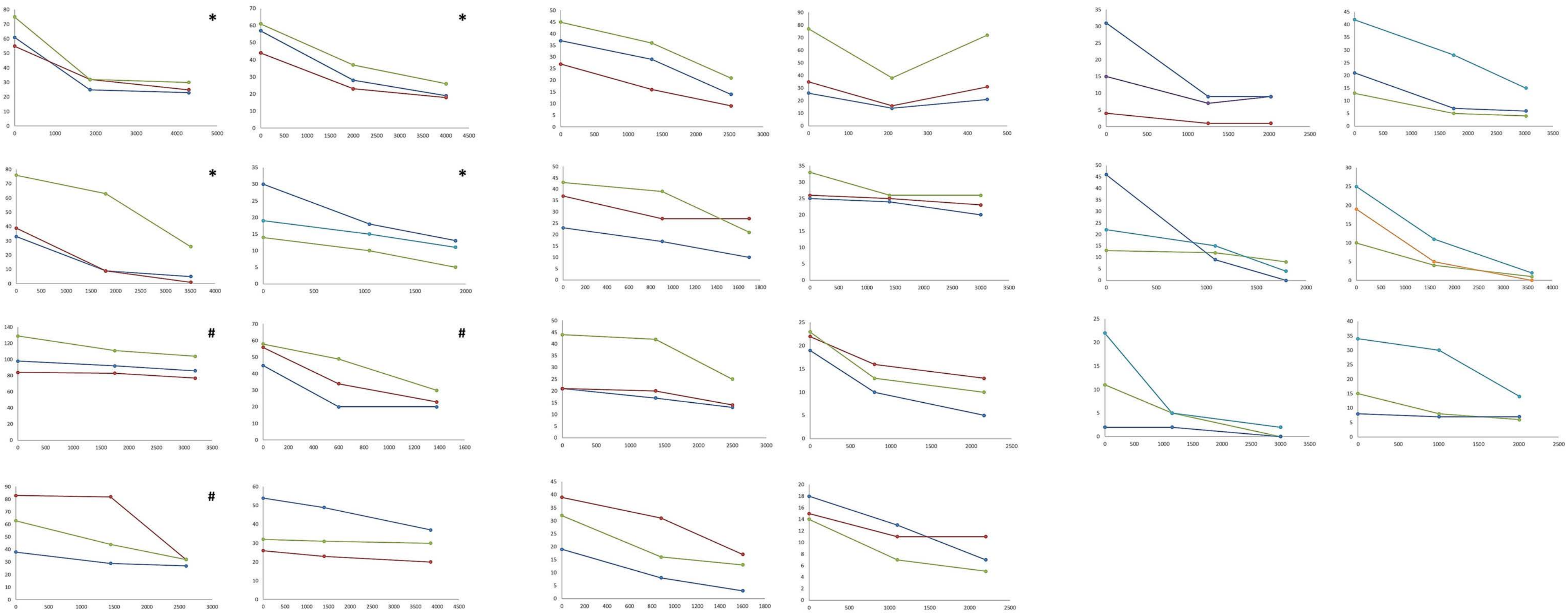

Supplement: Supplementary file 1 [file diagnostics-14-00589-s001.zip › Figure S2.pdf]
