# Supplementary material for: Lung Ultrasound Reproducibly Outperforms Computed Tomography in the Detection of Extravascular Lung Water in Patients Undergoing Haemodialysis
Source: Diagnostics (Basel). 2024 Mar 11;14(6):589. doi: 10.3390/diagnostics14060589 (PMC10969609; doi:10.3390/diagnostics14060589)
Supplement: Supplementary file 1 [file diagnostics-14-00589-s001.zip › Supplement Figure S1. LUS exam protocol.pdf]

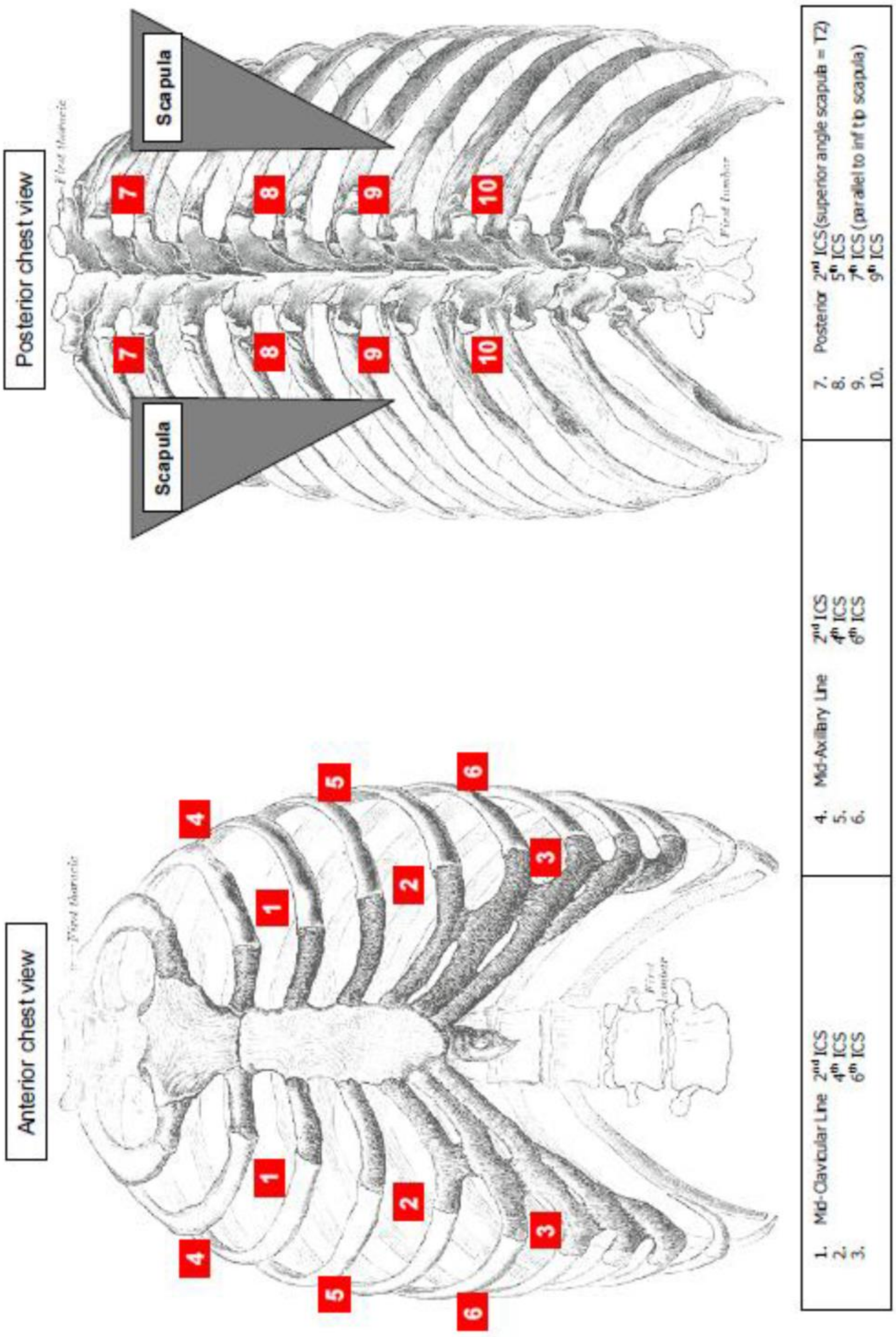

Figure S1. LUS exam protocol.

6-10 second clips to be acquired at 10 points across each hemithorax as above
