# Supplementary material for: Lung Ultrasound Reproducibly Outperforms Computed Tomography in the Detection of Extravascular Lung Water in Patients Undergoing Haemodialysis
Source: Diagnostics (Basel). 2024 Mar 11;14(6):589. doi: 10.3390/diagnostics14060589 (PMC10969609; doi:10.3390/diagnostics14060589)
Supplement: Supplementary file 1 [file diagnostics-14-00589-s001.zip › Supplement File S1. Participant VAS LUS Questionnaire.pdf]

OXFORD RESPIRATORY TRIALS UNIT

Oxford University Hospitals NHS Trust

UNIVERSITY OF OXFORD

Patient Study Number:			

Patient's DOB (mm/yyyy only)	
MM	YYYY

"A NEW WAY OF LOOKING AT YOUR LUNGS"

THORACIC ULTRASONOGRAPHY IN THE ASSESSMENT OF EXTRAVASCULAR LUNG WATER & RESPIRATORY FAILURE

REC REFERENCE: 13/SC/0319 CLINICALTRIALS.GOV IDENTIFIER: NCT01949402

PARTICIPANT VAS & ULTRASOUND SURVEY FORM (HAEMODIALYSIS STUDY ARM)

1. How breathless have you felt on average in the 24 hours before your dialysis today?

Not breathless at all |————————————————| Worst possible breathlessness

2. How breathless did you feel on average after your dialysis today?

Not breathless at all |————————————————| Worst possible breathlessness

3. Which statement best describes any change you have noticed in your breathing after your dialysis today? Please tick one box only.

- ☐ *My breathing feels significantly worse*
- ☐ *My breathing feels moderately worse*
- ☐ *My breathing feels slightly worse*
- ☐ *I have not noticed any change in my breathing*
- ☐ *My breathing feels slightly better*
- ☐ *My breathing feels moderately better*
- ☐ *My breathing feels significantly better*

4. How painful did you find the ultrasound scan(s) that you had today?

Not painful at all |————————————————| Worst possible pain

5. How time consuming did you find the ultrasound scan(s) that you had today? Please tick one box only.

- ☐ *Very time consuming*
- ☐ *Slightly time consuming*
- ☐ *Not time consuming at all*

2 COPIES – 1 (ORIGINAL) to be sent to ORTU and 1 (COPY) to be filed in patient's notes

Patient Study Number:			

Patient's DOB (mm/yyyy only)	
MM	YYYY

6. Did the doctor performing the ultrasound scan(s) today give you a clear explanation of what they were looking for?

- ☐ YES
- ☐ NO

7. Did you understand what the ultrasound scan(s) being done today were looking for? Please tick <u>one</u> box only.

- ☐ *I understood everything about what the ultrasound scan(s) were looking for.*
- ☐ *I understood most things about what the ultrasound scan(s) were looking for.*
- ☐ *I understood a little bit about what the ultrasound scan(s) were looking for.*
- ☐ *I did not understand anything about what the ultrasound scan(s) were looking for.*

8. Did you want to be given more information about what the ultrasound scan(s) being done today were looking for?

- ☐ YES
- ☐ NO

9. Would you be willing to have the same ultrasound scan(s) that you had today in the future?

- ☐ YES
- ☐ NO

10. Do you have any other comments about your experience today?

2 COPIES – 1 (ORIGINAL) to be sent to ORTU and 1 (COPY) to be filed in patient's notes
